# Negative Impact of Sigma-1 Receptor Agonist Treatment on Tissue Integrity and Motor Function Following Spinal Cord Injury

**DOI:** 10.3389/fphar.2021.614949

**Published:** 2021-02-10

**Authors:** Alise Lattard, Gaëtan Poulen, Sylvain Bartolami, Yannick N. Gerber, Florence E. Perrin

**Affiliations:** ^1^ MMDN, University of Montpellier, EPHE, INSERM, Montpellier, France; ^2^ Department of Neurosurgery, CHU, Montpellier, France; ^3^ Institut Universitaire de France (IUF), University of Montpellier, INSERM U1198, Montpellier, France

**Keywords:** spinal cord injury, sigma 1 receptor, PRE-084, motor function, neuromuscular junction, gliosis

## Abstract

In traumatic spinal cord injury, the initial trauma is followed by a cascade of impairments, including excitotoxicity and calcium overload, which ultimately induces secondary damages. The sigma-1 receptor is widely expressed in the central nervous system and is acknowledged to play a key role in calcium homeostasis. Treatments with agonists of the sigma-1 receptor induce beneficial effects in several animal models of neurological diseases. In traumatic injury the use of an antagonist of the sigma-1 receptor reversed several symptoms of central neuropathic pain. Here, we investigated whether sigma-1 receptor activation with PRE-084 is beneficial or detrimental following SCI in mice. First, we report that PRE-084 treatment after injury does not improve motor function recovery. Second, using *ex vivo* diffusion weighted magnetic resonance imaging completed by histological analysis, we highlight that σ1R agonist treatment after SCI does not limit lesion size. Finally, PRE-084 treatment following SCI decreases NeuN expression and increases astrocytic reactivity. Our findings suggest that activation of sigma-1 receptor after traumatic spinal cord injury is detrimental on tissue preservation and motor function recovery in mice.

## Introduction

Traumatic spinal cord injury (SCI) results in 0.6–0.9 million annual new cases worldwide (Kumar et al., 2018). SCI symptoms include sensory, motor and autonomic deficits ranging from minimal dysfunctions to complete tetraplegia. Following the primary traumatic mechanical disruption of spinal cord tissues, a cascade of events including vascular impairment, mitochondrial dysfunction, excitotoxicity and calcium overload characterizes the secondary phase of injury and further exacerbates the lesion. There is currently no curative treatment to improve neurological recovery after SCI.

The sigma-1 (σ1) receptor is a unique non-G-protein-coupled membrane-associated protein ubiquitously expressed with chaperone activity (Hayashi and Su, 2007). The central nervous system (CNS) is one of the major sites of σ1R activity. Indeed, σ1R is expressed in neurons, astrocytes, oligodendrocytes and microglia and is found at the endoplasmic reticulum (ER) membrane that is associated with the mitochondria (Mitochondria-Associated ER Membranes [MAMs]) [for review see (Penke et al., 2018)]. σ1R, that forms a complex at MAMs with BiP (binding immunoglobulin protein), another chaperone protein, is a Ca^2+^ sensitive and ligand-operated receptor chaperone at MAMs (Hayashi and Su, 2007). In fact, σ1R modulates Ca^2+^ exchange between ER and mitochondria by interacting with inositol-1,4,5 triphosphate receptors (IP3Rs) (Hayashi and Su, 2007). Following ligand stimulation, σ1R dissociates from BiP, conducting to a lengthened Ca^2+^ signaling into mitochondria. Decreasing σ1R activity in cell enhances apoptosis, whereas increasing it neutralizes ER stress response (Hayashi and Su, 2007). σ1R is amongst others implicated in neuroplasticity, neuroprotection and carcinogenesis (Hayashi and Su, 2007). Interestingly, newborn σ1R knockout mice, display a transient enhanced proliferation of progenitor cells in the hippocampal dentate gyrus followed by a decrease in survival and neurite outgrowth of newly generated neurons associated with a reduced function of N-methyl-D-aspartate receptor (NMDAr) (Sha et al., 2013).

PRE-084 (2-(4-morpholinoethyl)-1-phenylcyclohexane-1-carboxylate hydrochloride) is a highly selective σ1R agonist displaying minimal cross reactivity with other receptors [for extensive review see (Motawe et al., 2020)]. It had been shown *in vivo* that PRE-084 binds with σ1R and is rapidly distributed in the CNS (Motawe et al., 2020). Using excitotoxic injury in organotypic spinal cord slices, Guzmán-Lenis et al. demonstrated that PRE-084 not only induces a neuroprotective effect and decreases neuronal damage but also enhances axonal re-growth (Guzman-Lenis et al., 2009). Furthermore, σ1R activation attenuates several aspects of microglial activation in primary culture of rat microglia (Hall et al., 2009).

*In vivo*, the beneficial effects of PRE-084 has been reported in animal models of neurological diseases such as Alzheimer disease, Parkinson disease, amyotrophic lateral sclerosis, spinal muscular atrophy, Huntington disease, vascular dementia, amnesia, embolic stroke and multiple sclerosis. Though, PRE-084 did not improve outcomes following seizures [for review see (Motawe et al., 2020)].

To our knowledge, PRE-084 impact has not been investigate following acute traumatic injury in the CNS. However, it had been recently shown that after spinal cord contusion in mice, treatment with a σ1R antagonist reduced mechanical allodynia and thermal hyperalgesia that are characteristics of central neuropathic pain (Castany et al., 2018; Castany et al., 2019). On the one hand, treatment with *σ1*R agonist plays beneficial roles in chronic neurological diseases, including amelioration of motor-induced symptoms. On the other hand, treatment with *σ1*R antagonist induce positive outcomes on injury-induced pain following acute CNS injury. It is therefore of interest to investigate the role of σ1R activation on motor function recovery following SCI.

In this study, we assessed the effect of PRE-084 treatment following lateral hemisection of the mouse spinal cord on motor recovery and spinal cord structure at tissue and cellular levels.

## Material and Methods

### Study Approval

Experiments were approved by the French Ministry of National Education, Higher Education and Research, the regional ethic committee n°36 and by the Veterinary Services Department of Hérault (authorization n°34118) and followed the European legislative, administrative and statutory measures (EU/Directive/2010/63) and the ARRIVE guidelines.

### Spinal Cord Injury and Treatment

**FIGURE 1:**
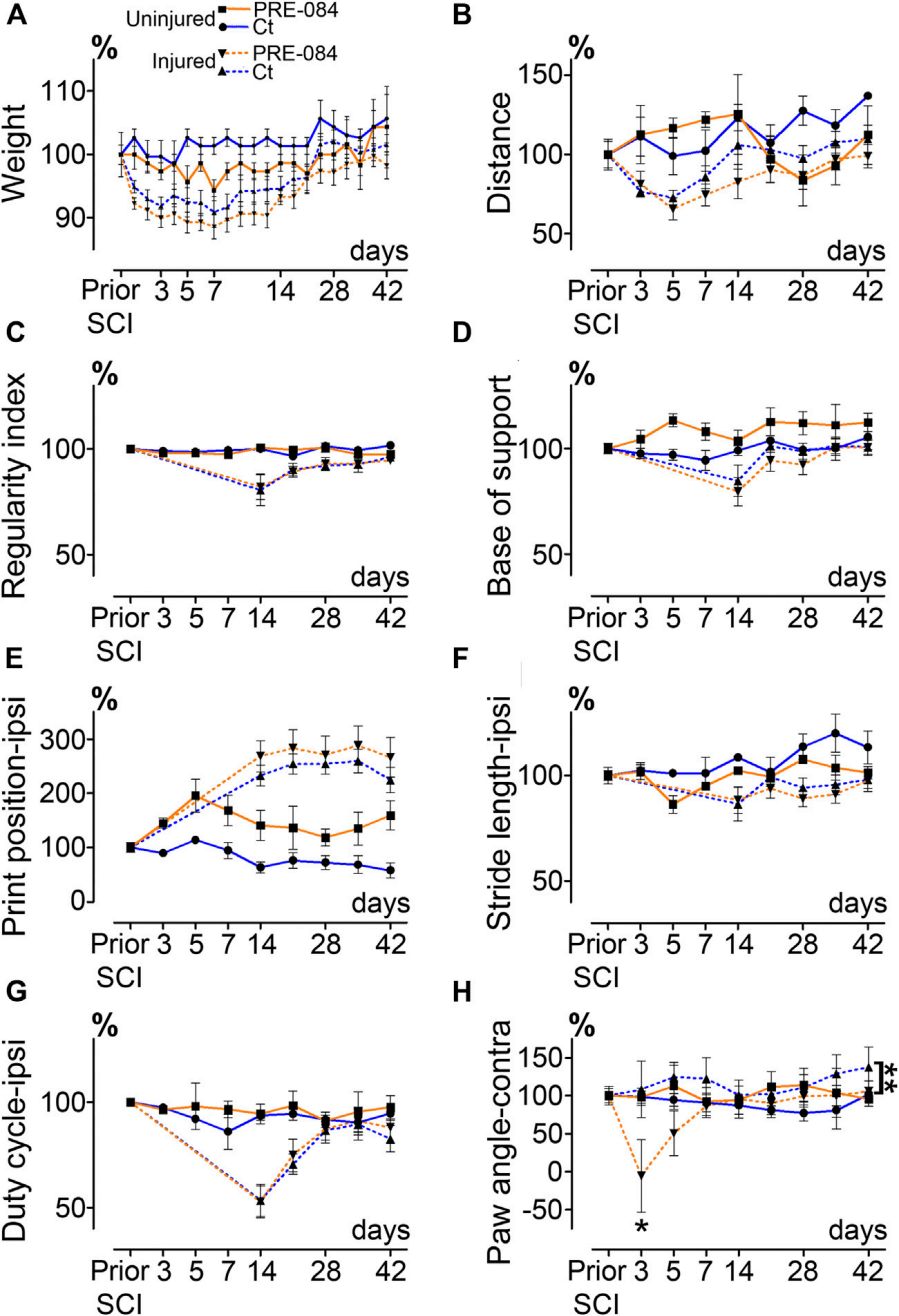
Effects of PRE-084 treatment on spontaneous motor activity and gait patterns after SCI. Weight curves **(A)**. Open field analysis of the distance covered in 8 min by mice in all groups **(B)**. Walking pattern analysis using CatWalk XT^®^ **(C–H)**. Parameters analyzed include the regularity index **(C)** and the base of support **(D)**. For paws located ipsilateral to the spinal cord lesion PRE-084 does not improve walking parameters such as the print position **(E)**, the stride length **(F)** and the duty cycle **(G)**. The paw angle of the hind paw located contralateral to the lesion is altered in the SCI-treated mice **(H)**. Number of mice: Uninjured mice 6 (3 treated and three untreated, plain lines), hemisected mice 24 (12 treated and 12 untreated, dashed lines). Statistics: two-way ANOVA followed by Bonferroni tests in the comparison between injured control and injured treated groups (dashed lines), **p < 0.01.

We used female mice of 5 months of age on a C57BL6/6J background (Charles River, Wilmington US) housed in controlled environment (hygrometry 60%, temperature 26–28°C, 12:12 light/dark cycle, food and water *ad libitum*). Following weaning females were housed in groups of 6. Three weeks prior surgery they were randomly affected to the untreated or treated groups. We used 3–4% isoflurane (Vetflurane^®^, Virbac, France) and 1 L/min oxygen flow rate to induce the anesthesia and then 1–2% isoflurane to maintain it throughout the surgery. We applied eye gel to the cornea at the beginning of the surgery. After shaving, the skin was cleaned (Vetadine^®^, Bayer, Australia). We performed a lateral spinal cord hemisection at thoracic 9 (T9) level to preserve complete respiratory function while obtaining a monoplegia. Hemisection was done using a micro knife (10315-12, Fine Science Tools) after vertebral laminectomy. Muscles and skin were sutured and mice were left to recover on a temperature-controlled pad. Post-operative cares: Animals were monitored over 1-h following the surgery before returning to their cages. Bladders were emptied manually twice daily until recovery of full sphincter control and observation of potential signs of pain or distress was carefully done. None of the animal developed self-biting, cutaneous infection or inflammation. We measured bodyweights prior to surgery and then daily until the study ends (Figure 1A). Treatment consisting in (2-(4-morpholinoethyl)-1-phenylcyclohexane-1-carboxylate hydrochloride) (PRE-084, Sigma-Aldrich, Saint-Quentin-Fallavier, France) diluted in the drinking water at a dose of 1 mg/kg/day, and given throughout 6 weeks after injury. To adjust and control the dose, we weighted the mice, we measured water consumption and we gave fresh PRE-084 solution twice a week.

Number of mice: six uninjured mice (three treated and three untreated) and 24 hemisected mice (12 treated and 12 untreated).

### Behavioral Analysis

All tests were done during the light cycle (from 1 pm to 4 pm). Open field test was always done first and followed by CatWalk test with a time interval of 1 h max. Habituation to tests was done to avoid stress-induced bias; mice were placed on the CatWalk XT^®^ glass plate as well as in the open field area for 20 min 7 days prior to the first recordings. Mice were then tested at seven- and 1-day prior injury followed by acquisitions at 72 h, 5 days and then once a week up to 6 weeks after lesion. Open field (EthoTrack, InnovationNet, Tiranges, France) and CatWalk XT^®^ (Noldus, Wageningen, Netherlands) were used to analyze statics and dynamics locomotor parameters as earlier described (Noristani et al., 2018). In brief, the open field test was used to assess spontaneous motor activity, animals were placed in an empty test arena (50 × 50 cm) and their spontaneous motor activity was video recorded. Each analysis session lasted 10 min (2 min without recording followed by 8 min video recorded). Dynamic walking patterns analysis was done using CatWalk XT^®^ through several parameters (including regularity index, base of support, print position, stride length, duty cycle and paw angle, Figures 1C–H). A minimum of five runs crossed at the same speed and at least 3-full step sequence patterns per run were analyzed. For injured mice, hind paw located on the ipsilateral side of SCI were not detectable by CatWalk before 14 days after injury, curves between 0- and 14-days post injury were thus extrapolated (Figures 1C–G).

### *Ex vivo* Diffusion Magnetic Resonance Imaging

**FIGURE 2:**
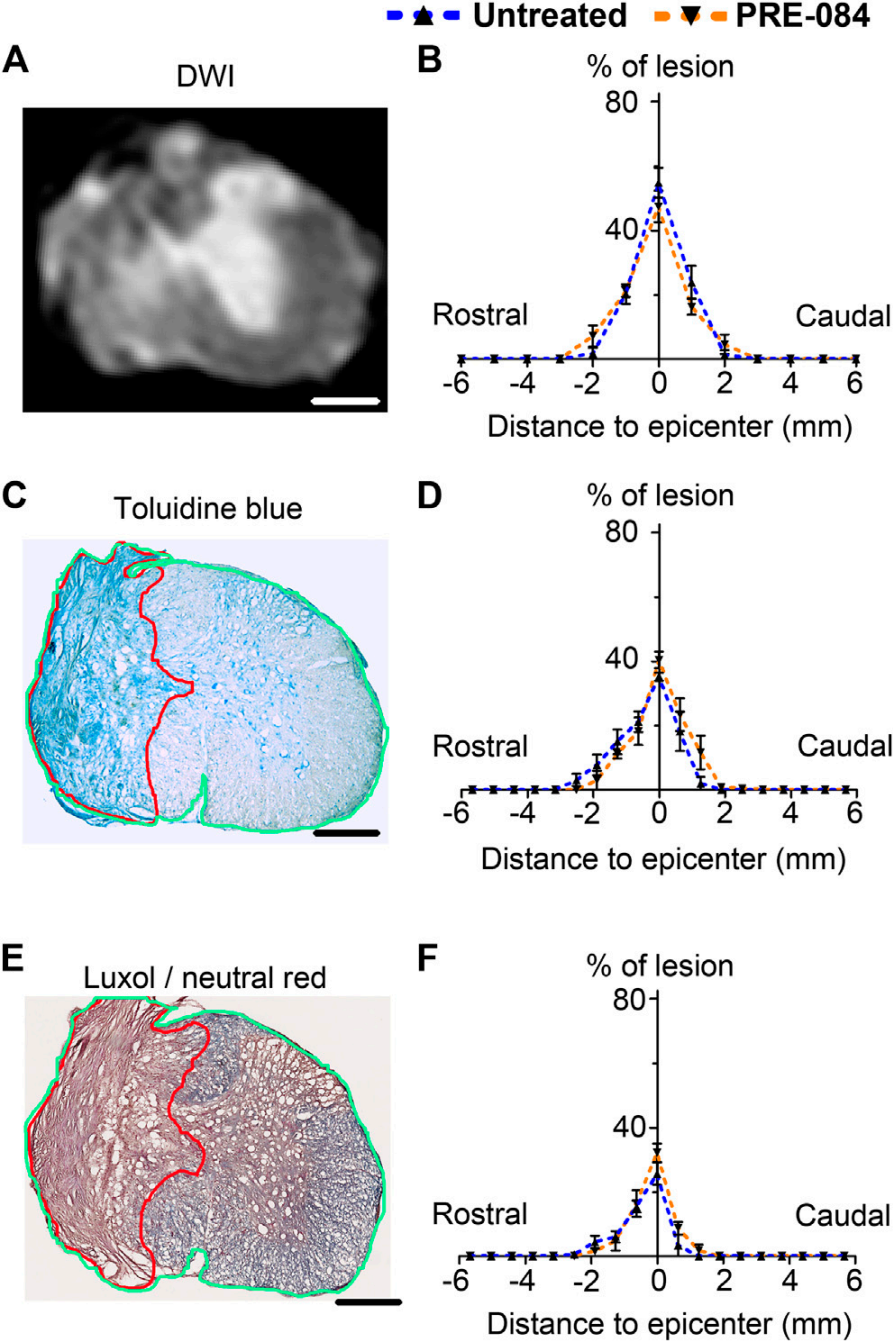
Lesion size analysis. Representative *ex vivo* diffusion weighted MRI (DWI) **(A)**, toluidine blue staining **(C)** and luxol fast blue/neutral red staining **(E)** at lesion epicenter 6 weeks after SCI. Quantifications of the lesion percentage at the epicenter, the lesion extension and the lesion volume represented by area under the curve using DWI acquisitions **(B)**, toluidine blue sections staining **(D)** and luxol fast blue/neutral red **(F)** in both treated and untreated injured animals. Note the damaged spinal cord tissue (outlined in red in **C** and **E**) used for lesion quantification and allows distinction from undamaged tissue (outlined in green in **C** and **E**) Number of hemisected mice: DWI six treated and six untreated and histology five treated and five untreated. Scale bars: **A, C and E** 600 μm.

Six weeks after SCI, mice were injected with a lethal dose of tribromoethanol (i.p., 500 mg/kg, Sigma-Aldrich Darmstadt, Germany) and perfused intracardially with phosphate saline buffer (PBS, 0.1M, pH 7.2) followed by 4% paraformaldehyde (PFA, pH 7.2, Sigma Aldrich, Darmstadt, Germany) in 0.1M PBS. Spinal cords were post-fixed for 2 h in 4% PFA and stored in PFA 1% until MRI acquisition. MRI acquisition and analysis were done as previously described (Noristani et al., 2018). Briefly, spinal cords were positioned in custom-made solenoid coil designed for spinal cord acquisition (Coillot et al., 2016) and placed in a 9.4 T apparatus (Agilent Varian 9.4/160/ASR, Santa Clara, California, USA). Diffusion MRI parameters: delta = 6.88 ms, G = 10 G/cm-1, separation = 15.05 ms, TR = 1,580 ms, TE = 30.55 ms, AVG = 30, FOV = 10 mm × 10 mm, slices = 36, thickness = 1 mm without gap and acquisition matrix = 128 × 128. Segmentations were done using Myrian software (Intrasense, Montpellier, France) to analyze the lesion area (% of the total surface area), the lesion extension along the rostro caudal axis and the lesion volume (area under the curve) (Figure 2B).

### Histology

Following *ex vivo* MRI, spinal cords were cryoprotected in 30% sucrose solution, frozen in OCT (Sakura, Alphen aan den Rijn, Netherlands) and stored at -20°C. Spinal cords were used for histological and immunohistochemical analysis on 14 μm thick axial cryosections as earlier described (Noristani et al., 2017). To confirm lesion extension, spinal cord sections were analyzed using toluidine blue and luxol fast blue/neutral red. Briefly, for toluidine blue staining sections were washed twice in 0.1 M PBS then incubated 10 min in 0.1% toluidine blue (Sigma Aldrich, Gilligham, United Kingdom), then soaked 10 s in 100% ethanol and washed twice in xylene. For luxol fast blue/neutral red staining, spinal cord sections were placed 5 min in 95% ethanol and then incubated in 0.1% luxol fast blue (12 h, room temperature, mild shaking). Slides were soaked 1 min in milli Q water before to be placed for 1 min in 0.05% lithium carbonate and washed 1 min in tap water. Slides were further incubated 10 min in 0.5% neutral red solution, dehydrated 5 min in 100% ethanol and washed twice 10 min in xylene. For both staining, slides were coverslipped with Eukitt (Sigma Aldrich, Gilligham, United Kingdom) and imaged using NanoZoomer Digital Pathology (NDP) System Hamamatsu (Hamamatsu, Hamamatsu city, Japan). On both rostral and caudal sides of the lesion, 10 sections were analyzed at 630 μm intervals using NDP view software. The lesion area was measured as % of the total surface area, lesion volume was estimated by the area under the curve (Figures 2D,F).

Peroxidase immunohistochemistry was performed as described before using anti-glial fibrillary acidic protein (GFAP) (1:1,000, catalog number Z0334, Dako, Glostrup, Denmark), anti-IBA1 (1:1,000, catalog number 019-19741, Wako Pure Chemical Industries, Osaka, Japan), anti-TMEM 119 (1:5, generous gift from Ben Barres's lab Stanford University, California, USA) and anti-NeuN (1:400, catalog number MAB 377, Merck Millipore, USA) primary antibodies and corresponding peroxidase-conjugated or biotinylated-conjugated (for TMEM119) secondary antibodies (1:500, Jackson Immunoresearch, Stratech Scientific Ltd, Soham, United Kingdom). Amplification was done for TMEM 119 staining through addition of Avidin Biotin Complex solution (Vector Laboratories Ltd. Peterborugh, United Kingdom) diluted at 1:100 in 0.1M PBS and incubated for 1 h at room temperature (RT). Sections were then rinsed in TRIS, pH 7.6 (Sigma-Aldrich Darmstadt, Germany) and protein expression was visualized using DAB peroxidase substrate kit (Vector Labs, Burlingame, USA). Nickel was added to DAB solution for TMEM119 staining. The reaction was stopped by 3 × 10 min rinses in 0.1M TRIS. For immunohistochemical experiments, up to 18 sections per mouse were analyzed (630 μm intervals throughout a 1.2 cm spinal cord segment centered on the lesion site). Peroxidase immunostainings were done at the same time for all animals for a given protein. Staining intensities were evaluated by measuring relative optical density (OD) using ImageJ (National Institutes of Health, USA). For neuromuscular junction (NMJ) quantifications, 16 μm transverse cryosections of gastrocnemius-soleus-plantaris muscular complex were proceeded for NMJ labeling using cholinesterase staining (Karnovsky and Roots, 1964). NMJs were quantified in every three sections.

### Statistics

Two-way analysis of variance (ANOVA) with Bonferroni post hoc tests were used for behavioral analysis. Unpaired t-tests with Welch correction were used for MRI and histological analysis. Significance was accepted at *p* ≤ 0.05. Data were analyzed using GraphPad Prism 5.0 (GraphPad Software, Inc., CA, USA) and expressed as the mean ± standard error of the mean.

## Results

### σ1R Agonist Treatment After Spinal Cord Injury Does Not Improve Motor Function and Preservation of Neuromuscular Junction Density

**FIGURE 3:**
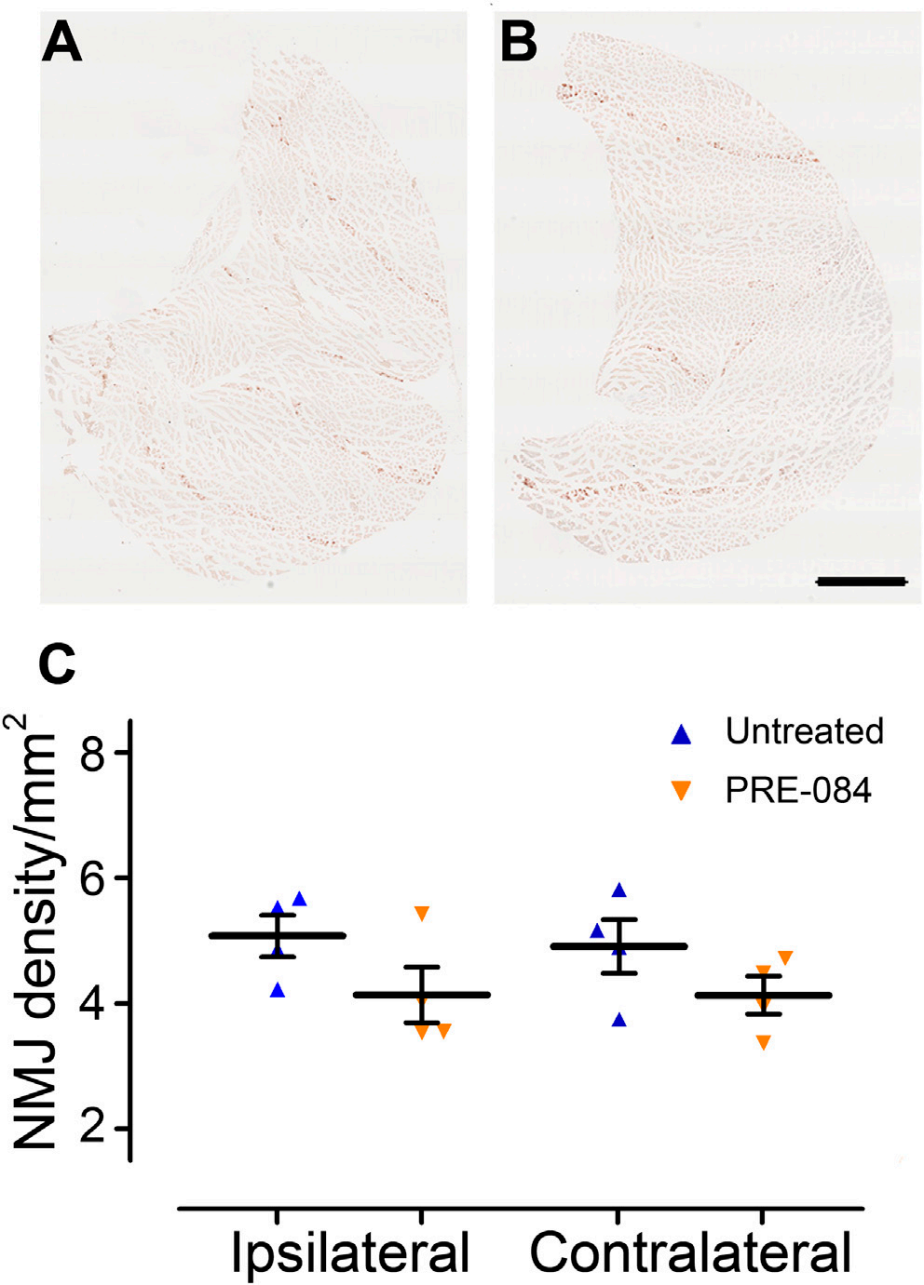
Effects of PRE-084 treatment on the overall neuromuscular junction density. *Gastrocnemius–soleus–plantaris* muscle complex of the hind limb located on the ipsilateral side of the lesion in untreated **(A)** and PRE-084-treated **(B)** mice. Quantitative assessments of neuromuscular junctions’ density in *gastrocnemius–soleus–plantaris* muscular complexes in untreated and PRE-084-treated mice **(C)**. Each dot represents the mean of at least 15 sections counted per animal per muscle. Number of injured mice: four treated and four untreated. Scale bars **A and B** 1 mm.

To investigate whether activation of σ1receptor after SCI is beneficial to motor recovery, we administered an oral 6-weeks PRE-084-treatment (1 mg/kg/day) to mice starting immediately after thoracic nine lateral hemisection of the spinal cord. As a prerequisite we verified that all behavioral parameters were affected by the injury (Figures 1A–H). PRE-084 did not modify the weight curves over the course of the experiment neither in uninjured nor injured mice (Figure 1A). We studied locomotion of the animal over the treatment period. PRE-084 did not improve the general spontaneous motricity analyzed by open field in both uninjured and injured groups (Figure 1B). The regularity index that reflects the number of normal step sequence patterns relative to the total number of paw placements (Figure 1C) and the base of support of the hind paws (average width between the paws, Figure 1D) were not improved in injured mice treated with PRE-084. Similarly, σ1R agonist treatment had no effect on the print position of paws on the ipsilateral side of the spinal cord lesion (corresponding to the distance between the position of the hind paw and the position of the previously placed front paw in the same step cycle, Figure 1E). Also, the stride length and duty cycle on the ipsilateral side (indicating the stance duration as a percentage of the duration of the step cycle) remained unchanged with PRE-084 treatment (Figures 1F,G). On the contralateral side, σ1receptor activation transiently worsened contralateral hind paw placement since the paw angle (angle between the paw axis and the body axis) differed more from preoperative value (100% correspond to the angle prior to SCI) in the SCI-treated than in the SCI-untreated group (Figure 1H, *p* = 0.049). Muscle alterations can affect paw angle placement, we thus investigated the *gastrocnemius-soleus-plantaris* complex (Figure 3 and Supplementary Figure 1A). No difference in number of neuromuscular junction densities in between groups was observed (Figures 3A–C, ipsilateral *p* = 0.15; untreated: 5.07 ± 0.33; PRE-084: 4.14 ± 0.45 and contralateral *p* = 0.2; untreated: 4.91 ± 0.43; PRE-084: 4.13 ± 0.30), however when taking into account all sections that were quantified (and not only the mean per animal), PRE-084-treatment decreased the neuromuscular junction densities in both hind limbs (Supplementary Figure 1A, *p* < 0.0001 for both ipsilateral; untreated: 5.22 ± 0.11; PRE-084: 4.19 ± 0.12 and contralateral; untreated: 5.07 ± 0.11; PRE-084: 4.00 ± 0.09).

Therefore, σ1receptor activation over a 6-weeks period starting immediately after SCI is slightly detrimental to motor function recovery in mice.

### σ1R Agonist Treatment After SCI Decreases NeuN Expression and Increases Astrocytic Reactivity Without Limiting Lesion Size

**FIGURE 4:**
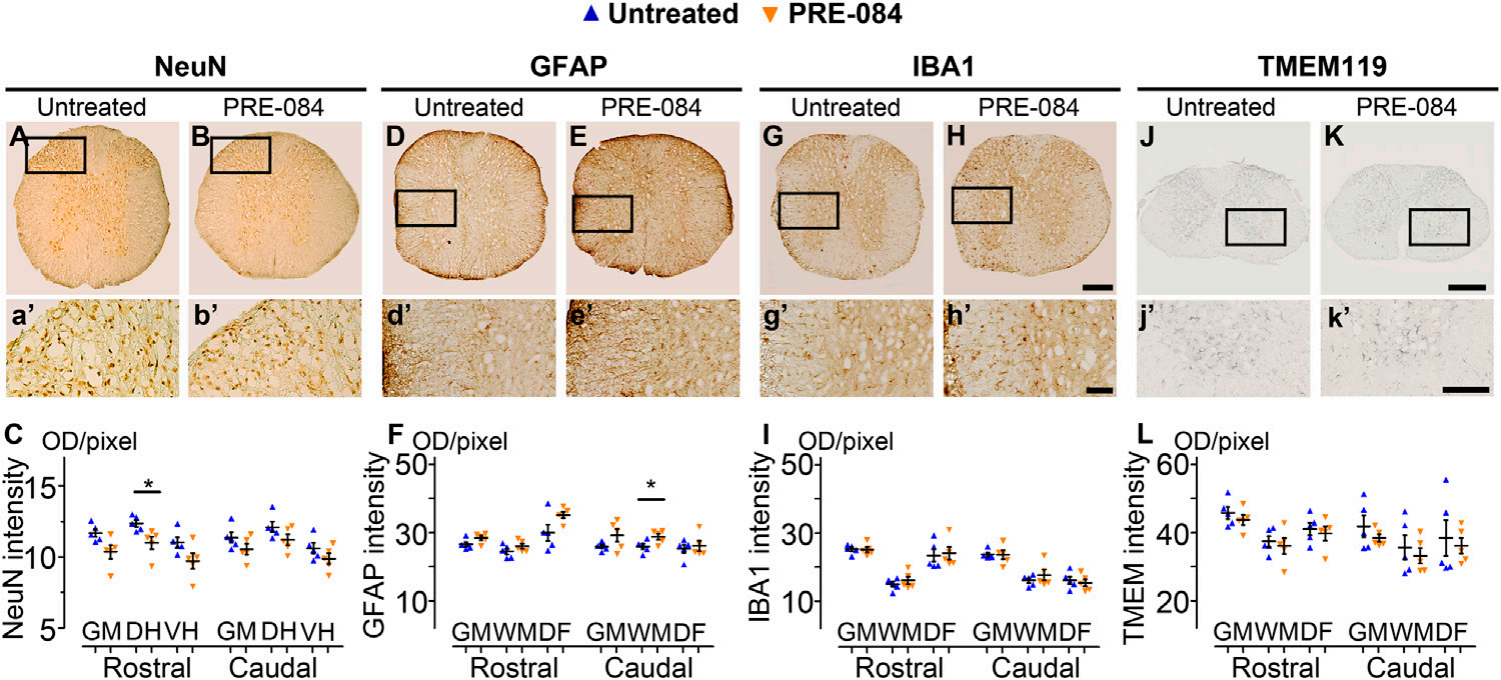
Histological analysis. NeuN-positive neurons in the gray matter 6 weeks after SCI in untreated (**A and a’**) and PRE-084-treated (**B and b’**) mice rostral to the lesion site. Quantification of NeuN-immunoreactivity in the gray matter, the dorsal and the ventral horns, rostral and caudal to the lesion **(C)**. GFAP-positive astrocytes 6 weeks after SCI in untreated (**D and d’**) and PRE-084-treated (**E and e’**) mice rostral to the lesion site. Quantification of GFAP-immunoreactivity in the gray matter, the white matter (excluding the dorsal *funiculus*) and the dorsal *funiculus* rostral and caudal to the lesion **(F)**. IBA1-positive microglia/macrophage 6 weeks after SCI in untreated (**G and g’**) and PRE-084-treated (**H and h’**) animals rostral to the lesion site. Quantification of IBA1-immunoreactivity in the gray matter, the white matter (excluding the dorsal *funiculus*) and the dorsal funiculus, rostral and caudal to the lesion **(I)**. TMEM 119-positive microglia 6 weeks after SCI in untreated (**J and j’**) and PRE-084-treated (**K and k’**) animals caudal to the lesion site. Quantification of TMEM119-immunoreactivity in the gray matter, the white matter (excluding the dorsal funiculus) and the dorsal *funiculus* rostral and caudal to the lesion (**L**). Higher magnifications (**a’-b’, d’-e’, g’-h’** and **j’-k’**) of black insets in **(A–B, D-E, G-H** and **J-K)**. In all graphs, results for untreated mice are in blue and PRE-084-treated are in orange. Data are expressed as mean ± SEM per group. Statistics: Student unpaired *t*-test with Welch correction (**C**, **F**, **I and L**), *p < 0.05. Scale bars: **A and B, D and E, G and H** 600 μm; **J and K** 500 μm; **a’ and b’, d’ and e’, g’ and h’** and **j’ and k’** 200 μm. GM: gray matter; VH: ventral horn; DH: dorsal horn; WM: white matter; DF: dorsal *funiculus*; OD = optical density. Each dot represents the mean of at least nine sections counted per animal per condition (630 μm interval between each section). Number of injured mice: five treated and five untreated.

To analyze outcomes of *σ*1receptor activation on tissue structure following spinal cord lesion we acquired *ex vivo* DW-MRI (Figure 2A) and segmented 1 mm-thick axial slices. Lesion extension on the rostro-caudal axis, lesion volume and percentage of damaged tissues at lesion epicenter were similar in both groups (Figure 2B). We further assessed the effect of PRE-084 on spinal cord lesion size using classical histological methods such as toluidine blue (Figures 2C,D) and luxol fast blue/neutral red (Figures 2E,F) staining on the same spinal cord segment and confirmed the absence of difference between untreated and treated groups (Figures 2D,F). We then deepened our analysis and quantified the expression of neuronal (Figures 4A,B and a’&b’) and glial markers (Figures 4D,E and d’&e’; G&H and g’&h’; J&K and j’&k’) on a 1.2 cm-perilesional segment of the spinal cord using specific neuron (NeuN), astrocytes (GFAP), microglia and macrophages (IBA1) and microglia (TMEM 119) (Figures 4A–K) markers. We highlighted a decrease in NeuN expression in the treated group as compared to the untreated group in the dorsal horn of the rostral segment (*p* = 0.032; untreated: 12.37 ± 0.23; PRE-084: 11.00 ± 0.43) (Figure 4C), though when taking into account all sections that were quantified (and not only the mean per animal), PRE-084-treatment decreased NeuN expression in all regions (gray matter, ventral and dorsal horns in the rostral (*p* < 0.0001) and caudal (*p* < 0.05) segments (Supplementary Figure 1B). GFAP expression was higher in the white matter of the treated group caudal to the lesion (*p* = 0.048; untreated: 25.94 ± 0.82; PRE-084: 28.79 ± 0.85) (Figure 4F), similarly when taking into account all sections that were quantified, PRE-084-treatment increased GFAP expression in all regions rostral to the lesion (*p* < 0.0001) and in the gray and the white matters (with the exception of the dorsal *funiculus*) caudal to the injury (Supplementary Figure 1C, *p* < 0.0001). No modulation of microglia/macrophages was induced by σ1R agonist treatment. The overall IBA1 (Figure 4I and Supplementary Figure 1D) and TMEM119 (Figure 4L and Supplementary Figure 1E) expressions were similar in both groups.

Altogether, these results suggest that activation of σ1 receptor using PRE-084 over 6 weeks after injury induces a decrease in NeuN expression and an increase in astrogliosis while microglia reactivity is not affected.

## Discussion

In this study, we assessed outcomes of sigma-1 receptor agonist treatment on functional recovery and tissue reorganization after SCI in mice. Our findings suggest a detrimental effect of PRE-084 post injury treatment on motor function recovery and is associated, at tissue level, with a decrease in NeuN expression and an increase in astrogliosis.

The involvement of σ1R in central neuropathic pain induced by SCI had been recently studied using both genetic and pharmacological approaches (Castany et al., 2018; Castany et al., 2019). Following spinal cord contusion at thoracic level 8–9 mice lacking σ1R (σ1KO) display reduced injury-induced thermal hyperalgesia and mechanical allodynia. Moreover, σ1KO do not present an injury-induced increase in spinal cord expression of the phosphorylated form of two molecules involved in central sensitization in neuropathic pain states i.e. extracellular signal-regulated kinases (ERK1/2) and NMDA receptor NR2B subunit. Conversely to wild type mice, SCI in σ1KO mice does not induce an upregulation of the pro inflammatory cytokines tumor necrosis factor (TNF-α) and interleukin IL-1β (Castany et al., 2018). Mice treated with a selective σ1R antagonist starting either 1 month (Castany et al., 2018) or 1 week (Castany et al., 2019) following SCI, similarly display reduced induced-central neuropathic pain. No major modification of motor activity was observed in both treatment protocols. Moreover, σ1R antagonist treatment during the first week post SCI also prevented injury-induced increases of pERK1/2, pNR2B-NMDA, TNF-α and IL-1β (Castany et al., 2019). In order to implement the two previous studies that investigated outcomes of a σ1R pharmacological modulation following SCI, we have chosen to perform our experiments in female mice. It had been shown a gender-dependent locomotor recovery following SCI; thus we cannot exclude a differential effect of sigma one agonist treatment in male mice (Farooque et al., 2006). Our findings, further suggest a detrimental effect of σ1R activation following SCI on motor function recovery that may thus result from an increased expression of proinflammatory molecules as reflected by an enhanced astrogliosis.

Sigma-1 receptor modulates calcium signaling between the endoplasmic reticulum and mitochondria and regulates mitochondrial functions, such as intramitochondrial Ca^2+^ homeostasis, reactive oxygen species (ROS) generation and cellular bioenergetics (Hayashi and Su, 2007). Mitochondrial dysfunction is an essential component of the secondary injury cascade induced by SCI. In particular, it leads to ATP loss and successive inactivation of ATP-dependent ion pumps required for regulation of ion concentrations and reuptake of the excitatory neurotransmitter glutamate. Moreover, a consequence of the persistent ion shift during secondary injury is an increased ROS. On the one hand, sigma-1 receptor agonists favor brain plasticity and induce protective effect in the CNS under pathological conditions (Maurice and Goguadze, 2017) but on the other hand isolated mouse brain mitochondria exposed to σ1R agonists display an increase in ROS level. Strikingly, PRE-084 induces a ROS increase of 24% associated with a 40% selective increase of complex one activity when applied to isolated mouse brain mitochondria (non-pathological condition) and decreases Aβ1–42-induced ROS elevation (pathological condition i.e. amyloid toxicity) (Goguadze et al., 2019). Therefore, σ1R agonists exert condition-dependent effects on ROS production. The detrimental *in vivo* effect of *per os* PRE-084 treatment over 6 weeks at this dose (1 mg/kg/day) following lateral hemisection of the spinal cord may thus be a consequence of an increased ROS level.

The present study therefore suggests the detrimental effect of σ1R agonist on motor recovery, neuronal preservation and astrogliosis following SCI. However, dose-dependent and time response effects of σ1R agonists and antagonists treatments in various animal models of traumatic CNS injury merits further investigation.

## Data Availability

The raw data supporting the conclusions of this article will be made available by the authors, without undue reservation.
